# Parameterized Complexity of Directed Spanner Problems

**DOI:** 10.1007/s00453-021-00911-x

**Published:** 2021-12-27

**Authors:** Fedor V. Fomin, Petr A. Golovach, William Lochet, Pranabendu Misra, Saket Saurabh, Roohani Sharma

**Affiliations:** 1 Department of Informatics, University of Bergen, PB 7803, 5020 Bergen, Norway; 2 Max Planck Institute for Informatics, Saarland Informatics Campus, Saarbrücken, Germany; 3 Institute of Mathematical Sciences, HBNI, Chennai, India

**Keywords:** Graph spanners, Directed graphs, Parameterized complexity, Kernelization

## Abstract

We initiate the parameterized complexity study of minimum *t*-spanner problems on directed graphs. For a positive integer *t*, a multiplicative *t*-spanner of a (directed) graph *G* is a spanning subgraph *H* such that the distance between any two vertices in *H* is at most *t* times the distance between these vertices in *G*, that is, *H* keeps the distances in *G* up to the distortion (or stretch) factor *t*. An additive *t*-spanner is defined as a spanning subgraph that keeps the distances up to the additive distortion parameter *t*, that is, the distances in *H* and *G* differ by at most *t*. The task of Directed Multiplicative Spanner is, given a directed graph *G* with *m* arcs and positive integers *t* and *k*, decide whether *G* has a multiplicative *t*-spanner with at most $m-k$ arcs. Similarly, Directed Additive Spanner asks whether *G* has an additive *t*-spanner with at most $m-k$ arcs. We show that (i) Directed Multiplicative Spanner admits a polynomial kernel of size $\mathcal {O}(k^4t^5)$ and can be solved in randomized $(4t)^k\cdot n^{\mathcal {O}(1)}$ time, (ii) the weighted variant of Directed Multiplicative Spanner can be solved in $k^{2k}\cdot n^{\mathcal {O}(1)}$ time on directed acyclic graphs, (iii) Directed Additive Spanner is ${{\,\mathrm{\mathsf{W}}\,}}[1]$-hard when parameterized by *k* for every fixed $t\ge 1$ even when the input graphs are restricted to be directed acyclic graphs. The latter claim contrasts with the recent result of Kobayashi from STACS 2020 that the problem for undirected graphs is ${{\,\mathrm{\mathsf{FPT}}\,}}$ when parameterized by *t* and *k*.

## Introduction

Given a (directed) graph *G*, a *spanner* is a spanning subgraph of *G* that approximately preserves distances between the vertices of *G*. Graph spanners were formally introduced by Peleg and Schäffer in [16] (see also [17]). Originally, the concept was introduced for constructing network synchronizers [17]. However, graph spanners have a plethora of theoretical and practical applications in various areas like efficient routing and fast computing of shortest paths in networks, distributed computing, robotics, computational geometry and biology. We refer to the recent survey of Ahmed et al. [1] for the introduction to graph spanners and their applications.

We are interested in the classical *multiplicative* and *additive* graph spanners in graphs. Let *G* be a (directed) graph. For two vertices $u,v\in V(G)$, ${{\text {dist}}}_G(u,v)$ denotes the *distance* between *u* and *v* in *G*, that is, the number of edges (arcs, respectively, for the directed case) of a shortest (*u*, *v*)-path. Let *t* be a positive integer. It is said that a spanning subgraph *H* of *G* is a *multiplicative t-spanner* if ${{\text {dist}}}_H(u,v)\le t\cdot {{\text {dist}}}_G(u,v)$ for every two vertices $u,v\in V(G)$, i.e., *H* approximates distances in *G* within factor *t*. A spanning subgraph *H* of *G* is called an *additive t-spanner* if ${{\text {dist}}}_H(u,v)\le {{\text {dist}}}_G(u,v)+t$ for every $u,v\in V(G)$, that is, *H* approximates the distances in *G* within the additive parameter *t*. The standard task in the graph spanner problems is, given an allowed distortion parameter *t*, find a sparsest *t*-spanner, i.e., a spanner with the minimum number of edges. We consider the parameterized versions of this task:
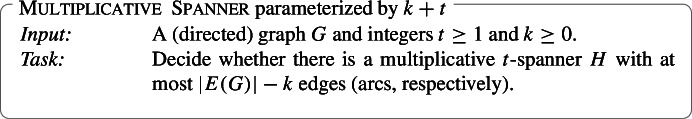


and
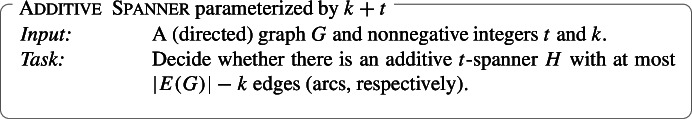


Informally, the task of these problems is to decide whether we can delete at least *k* edges (arcs, respectively, for the directed case) in such a way that all the distances in the resulting graph are “*t*-close” to the original ones.

*Previous work.* We refer to [1] for the comprehensive survey of the known results and mention here only those that directly concern our work. First, we point that the considered graph spanner problems are computationally hard. It was already shown by Peleg and Schäffer in [16] that deciding whether an undirected graph *G* has a multiplicative *t*-spanner with at most $\ell$ edges is ${{\,\mathrm{\mathsf{NP}}\,}}$-complete even for fixed $t=2$. In fact, the problem is ${{\,\mathrm{\mathsf{NP}}\,}}$-complete for every fixed $t\ge 2$ [2]. Moreover, for every $t\ge 2$, it is ${{\,\mathrm{\mathsf{NP}}\,}}$-hard to approximate the minimum number of edges of a multiplicative *t*-spanner within the factor $c\log n$ for some $c>1$ [12]. The same complexity lower bounds for directed graphs were also shown by Cai [2] and Kortsarz [12]. Stronger inapproximability bounds were given by Elkin and Peleg [7]. Additive *t*-spanners for undirected graphs were introduced by Liestman and Shermer in [13, 14]. In particular, they proved in [14], that for every fixed $t\ge 1$, it is ${{\,\mathrm{\mathsf{NP}}\,}}$-complete to decide whether a graph *G* admits an additive *t*-spanner with at most $\ell$ edges. It was shown by Chlamtác et al. [4] that for every integer $t\ge 1$ and any constant $\varepsilon >0$, there is no polynomial-time $2^{\log ^{1-\varepsilon } n}/t^3$-approximation for the minimum number of edges of an additive *t*-spanner unless ${{\,\mathrm{\mathsf{NP}}\,}}\subseteq \mathsf{DTIME}(2^\mathrm{polylog(n)})$.

The aforementioned hardness results make it natural to consider these spanner problems in the parameterized complexity framework. The investigation of Multiplicative Spanner and Additive Spanner on undirected graphs was initiated by Kobayashi in [10] and [11]. In [10], it was proved that Multiplicative Spanner admits a polynomial kernel of size $\mathcal {O}(k^2t^2)$. For Additive Spanner, it was shown in [11] that the problem can be solved in time $2^{\mathcal {O}((k^2+kt)\log t)}\cdot n^{\mathcal {O}(1)}$, that is, the problem is *fixed-parameter tractable* (${{\,\mathrm{\mathsf{FPT}}\,}}$) when parameterized by *k* and *t*.

*Our results.* We initiate the study of Multiplicative Spanner and Additive Spanner on directed graphs and further refer to them as Directed Multiplicative Spanner and Directed Additive Spanner, respectively. We show that Directed Multiplicative Spanner admits a kernel of size $\mathcal {O}(k^4t^5)$. We complement this result by observing that the problem can be solved in $(4t)^k\cdot n^{\mathcal {O}(1)}$ time by a Monte Carlo algorithm with false negatives. We leave open the question whether the problem is ${{\,\mathrm{\mathsf{FPT}}\,}}$ when parameterized by *k* only. However we show that this is the case when the inputs are restricted to be *directed acyclic graphs* (DAGs). In fact, we show the more general claim for the weighted variant of the problem called Weighted Directed Multiplicative Spanner, where the input directed graph *G* is supplied with a weight function $\omega (\cdot )$ with positive values defining the lengths of the arcs and the distances are defined with respect to these arc lengths. Clearly, Directed Multiplicative Spanner is the special case of this problem with unit arc lengths. We show that Weighted Directed Multiplicative Spanner can be solved in $k^{2k}\cdot n^{\mathcal {O}(1)}$ on DAGs. We also observe that Directed Multiplicative Spanner is ${{\,\mathrm{\mathsf{NP}}\,}}$-complete on DAGs. For additive spanners, we show that the problem becomes much harder on directed graphs by showing that Directed Additive Spanner is ${{\,\mathrm{\mathsf{W}}\,}}[1]$-hard for every *fixed* $t\ge 1$ even on DAGs.

*Organization of the paper.* In Section 2, we introduce basic notions used in the paper. In Section 3, we prove that Directed Multiplicative Spanner admits a polynomial kernel and sketch an ${{\,\mathrm{\mathsf{FPT}}\,}}$ algorithm. Further in this section, we show that Directed Multiplicative Spanner is ${{\,\mathrm{\mathsf{NP}}\,}}$-complete on DAGs and prove that Weighted Directed Multiplicative Spanner is ${{\,\mathrm{\mathsf{FPT}}\,}}$ for the parameterization by *k* only for this class of directed graphs. In Section 4, we show hardness for Directed Additive Spanner. We conclude in Section 5 by stating some open problems.

## Preliminaries

*Parameterized Complexity and Kernelization.* We refer to the recent books [5, 6, 8] for the detailed introduction. In the Parameterized Complexity theory, the computational complexity is measured as a function of the input size *n* of a problem and an integer *parameter* *k* associated with the input. A parameterized problem is said to be *fixed-parameter tractable* (or ${{\,\mathrm{\mathsf{FPT}}\,}}$) if it can be solved in time $f(k)\cdot n^{\mathcal {O}(1)}$ for some function $f(\cdot )$. A *kernelization * algorithm for a parameterized problem $\Pi$ is a polynomial algorithm that maps each instance (*I*, *k*) of $\Pi$ to an instance $(I',k')$ of $\Pi$ such that

(i) (*I*, *k*) is a yes-instance of $\Pi$ if and only if $(I',k')$ is a yes-instance of $\Pi$, and
(ii) $|I'|+k'$ is bounded by *f*(*k*) for a computable function $f(\cdot )$.

Respectively, $(I',k')$ is a *kernel* and $f(\cdot )$ is its *size*. A kernel is *polynomial* if $f(\cdot )$ is polynomial. It is common to present a kernelization algorithm as a series of *reduction rules*. A reduction rule for a parameterized problem is an algorithm that takes an instance of the problem and computes in polynomial time another instance that is more “simple” in a certain way. A reduction rule is *safe* if the computed instance is equivalent to the input instance.

*Graphs.* Recall that an undirected graph is a pair $G=(V,E)$, where *V* is a set of vertices and *E* is a set of unordered pairs $\{u,v\}$ of distinct vertices called *edges*. A directed graph $G=(V,A)$ is a pair, where *V* is a set of vertices and *A* is a set of ordered pairs (*u*, *v*) of distinct vertices called *arcs*. We say that *u* and *v* are *incident* to (*u*, *v*). Note we do not allow loops and multiple arcs (that are irrelevant for distances). We use *V*(*G*) and *E*(*G*) (*A*(*G*), respectively) to denote the set of vertices and the set of edges (set of arcs, respectively) of *G*. For a (directed) graph *G* and a subset $X\subseteq V(G)$ of vertices, we write *G*[*X*] to denote the subgraph of *G* induced by *X*. For a set of vertices *S*, $G-S$ denotes the (directed) graph obtained by deleting the vertices of *S*, that is, $G-S=G[V(G)\setminus S]$; for a vertex *v*, we write $G-v$ instead of $G-\{v\}$. Similarly, for a set of edges (arcs, respectively) *S* (an edge or arc *e*, respectively), $G-S$ ($G-e$, respectively) denotes the graph obtained by the deletion of the elements of *S* (the deletion of *e*, respectively). A subgraph *H* of a (directed) graph *G* is a *spanning subgraph* of *G* if $V(H)=V(G)$. Every directed acyclic graph (DAG) *G* has a topological ordering of its vertex set, that is there exists $\pi :V(G) \rightarrow \{1, \ldots , |V(G)|\}$ such that if $(u,v) \in A(G)$ then $\pi (u) < \pi (v)$. The notation $u \prec v$ denotes $\pi (u) < \pi (v)$ and $u \preceq v$ denotes that either $\pi (u) < \pi (v)$ or $u=v$.

We write $P=v_1\cdots v_k$ to denote a *path* with the vertices $v_1,\ldots ,v_k$ and the edges $\{v_1,v_2\},\ldots ,\{v_{k-1},v_k\}$ (arcs $(v_1,v_2),\ldots ,(v_{k-1},v_k)$, respectively); $v_1$ and $v_k$ are the *end-vertices* of *P* and we say that *P* is a $(v_1,v_k)$*-path*. A single vertex path is *trivial*, and for a trivial $P=v$, *P* is a (*v*, *v*)-path. All considered paths are assumed to be *simple*, that is, $v_1,\ldots ,v_k$ are distinct. The *length* of a path is the number of edges (arcs, respectively) in the path. Also *A*(*P*) denotes the arc set of the path *P*. For a (*u*, *v*)-path $P_1$ and a (*v*, *w*)-path $P_2$, we denote by $P_1\circ P_2$ the *concatenation* of $P_1$ and $P_2$. We use similar notation for walks; the difference between and a path and a walk is that, the vertices of a walk $W=v_1\cdots v_k$ are not required to be distinct and a walk may go through the same edges (arcs, respectively) several times. Notice that the concatenation of two paths is a walk but not necessarily a path. For two vertices $u,v\in V(G)$, ${{\text {dist}}}_G(u,v)$ denotes the *distance* between *u* and *v* in *G*, that is, the length of a shortest (*u*, *v*)-path; we assume that ${{\text {dist}}}_G(u,v)=+\infty$ if there is no (*u*, *v*)-path in *G*. Clearly, ${{\text {dist}}}_G(u,v)={{\text {dist}}}_G(v,u)$ for undirected graphs but this not always the case for directed graphs.

Let *t* be a positive integer. It is said that a spanning subgraph *H* of *G* is a *multiplicative t-spanner* if ${{\text {dist}}}_H(u,v)\le t\cdot {{\text {dist}}}_G(u,v)$ for every $u,v\in V(G)$. A spanning subgraph *H* of *G* is called an *additive t-spanner* if ${{\text {dist}}}_H(u,v)\le {{\text {dist}}}_G(u,v)+t$ for every $u,v\in V(G)$.

We also consider the weighted variant of spanners for directed graph. Let *G* be an *arc wighted* directed graph, that is, we a given a *weight* (or *length*) function $\omega :A(G)\rightarrow \mathbb {R}^+$ with positive values; we say that $\omega (e)$ is the *length* of an arc *e*. The *length* of a weighted path $P=v_1\cdots v_k$ is $\sum _{i=2}^k=\omega (v_{i-1},v_i)$; the length of a trivial path is zero. Then the *weighted distance* ${{\text {dist}}}_G^\omega (u,v)$ is the length of a shortest path with respect to the arc lengths. For real $t\ge 1$ and a weighted directed graph *G*, it is said that a spanning subgraph *H* a *weighted multiplicative t-spanner* if ${{\text {dist}}}_H^\omega (u,v)\le t\cdot {{\text {dist}}}_G^\omega (u,v)$ for every $u,v\in V(G)$; note that the stretch factor *t* is not required to be an integer.

## Directed Multiplicative *t*-spanners

In this section, we consider Directed Multiplicative Spanner. We show that the problem admits a polynomial kernel and then complement this result by obtaining an ${{\,\mathrm{\mathsf{FPT}}\,}}$ algorithm. Further, we consider multiplicative spanners on DAGs. We prove that Directed Multiplicative Spanner is ${{\,\mathrm{\mathsf{NP}}\,}}$-complete on this class of directed graphs and show that Weighted Directed Multiplicative Spanner is ${{\,\mathrm{\mathsf{FPT}}\,}}$ when parameterized by *k* only. These results are based on *locality* of multiplicative spanners in the sense of the following observation made by Peleg and Schäffer [16].

### Observation 1

([16]). Let *t* be a positive integer (or $t>1$ be a real for the weighted case). A spanning subgraph *H* of a directed graph *G* is a (weighted) multiplicative *t*-spanner if and only if for every arc $(u,v)\in A(G)$, there is a (*u*, *v*)-path in *H* of length at most *t* (*t* times the length of (*u*, *v*) in the weighted case).

Let *t* be a positive integer (or real for the weighted spanners) and let *G* be a directed graph. For an arc $a=(u,v)$ of *G*, we say that a (*u*, *v*)-path *P* is a *t-detour* for *a* if the length of *P* is at most *t* (*t* times the length of *a* in the weighted case) and *P* does not contain *a*. By Observation 1, to solve Directed Multiplicative Spanner for (*G*, *t*, *k*), it is necessary and sufficient to identify *k* arcs that have *t*-detours that do not contain selected arcs. Then *H* can be constructed by deleting these arcs. Notice that this observation holds for both unweighted and weighted spanners. However, for the weighted case, the number of arcs in a *t*-detour may be arbitrary and depends on the length of *a*.

### Polynomial Kernel for Directed Multiplicative Spanner

In this subsection, we show that Directed Multiplicative Spanner admits a polynomial kernel.

#### Theorem 1

Directed Multiplicative Spanner has a kernel of size $\mathcal {O}(k^4t^5)$.

#### Proof

Let (*G*, *t*, *k*) be an instance of Directed Multiplicative Spanner. Clearly, if $k=0$, then (*G*, *t*, *k*) is a yes-instance, and our algorithm returns a trivial yes-instance in this case. We assume from now that $k>0$.

We say that $a\in A(G)$ is *t-good* if *G* has a *t*-detour for *a*. Let *S* be the set of *t*-good arcs. Clearly, *S* can be constructed in polynomial time by making use of Dijkstra’s algorithm. We follow the idea of Kobayashi [10] for constructing a polynomial kernel for undirected case and show that if *S* is sufficiently big, then (*G*, *t*, *k*) is a yes-instance of Directed Multiplicative Spanner.

#### Claim 1

If $|S|\ge \frac{1}{2}k(t+1)((k-1)t+2)$, then (*G*, *t*, *k*) is a yes-instance of Directed Multiplicative Spanner.

#### Proof of Claim 1

Let $|S|\ge \frac{1}{2}k(t+1)((k-1)t+2)$. For every $a\in S$, let $P_a$ be a *t*-detour for *a*.

Let $S_0=\emptyset$. For $i=1,\ldots ,k$, we iteratively construct sets of arcs $S_1,\ldots ,S_k$ such that

$$\begin{aligned} S_0\subset S_1\subset \cdots \subset S_k\subseteq S \end{aligned}$$

and sets of arcs $R_i$ such that $R_i\subseteq S_i\setminus S_{i-1}$ and $|R_i|=(k-i)t+1$ for $i\in \{1,\ldots ,k\}$ using the following procedure. For $i=1,\ldots ,k$,

- select an arbitrary set $R_i$ of size $(k-i)t+1$ in $S\setminus S_{i-1}$,
- set $$S_i=S_{i-1}\cup \bigcup _{a\in R_i}\big ( (A(P_a)\cap S)\cup \{a\}\big )$$.

We show by induction, that the sets $S_1,\ldots ,S_k$ and $R_1,\ldots ,R_k$ exist. Since $|S\setminus S_0|=|S|\ge (k-1)t+1$, we conclude that $R_1$ of size $(k-1)t+1$ can be selected. Assume that the sets $S_j$ and $R_j$ have been constructed for $0\le j<i\le k$. Observe that because $|\bigcup _{a\in R_j}\big ((A(P_a)\cap S)\cup \{a\}\big )|\le (t+1)|R_j|$,

$$\begin{aligned} |S_j\setminus S_{j-1}|\le |R_j|(t+1)=((k-j)t+1)(t+1) \end{aligned}$$

for $$1\le j<i$$. Therefore,

1 $$\begin{aligned} |S_{i-1}|\le \sum _{j=1}^{i-1}(((k-j)t+1)(t+1)). \end{aligned}$$

Notice that

2 $$\begin{aligned} \frac{1}{2}k(t+1)((k-1)t+2)=\sum _{j=1}^k(((k-j)t+1)(t+1)). \end{aligned}$$

Then by (1) and (2),

$$\begin{aligned} |S\setminus S_{i-1}|\ge \sum _{j=i}^k(((k-j)t+1)(t+1))\ge (k-i)t+1. \end{aligned}$$

This means that $R_i$ can be selected and we can construct $S_i$.

Now we select arcs $a_i\in R_i$ for $i=k,k-1,\ldots ,1$. Since $|R_k|=1$, the choice of $a_k$ is unique. Assume that $a_k,\ldots ,a_{i+1}$ have been selected for $1< i+1\le k$. Then we select an arbitrary

$$\begin{aligned} a_i\in R_i\setminus \bigcup _{j=i+1}^k A(P_{a_j}). \end{aligned}$$

Because $|\bigcup _{j=i+1}^k A(P_{a_j})|\le (k-i)t$ and $|R_i|=(k-i)t+1$, $a_i$ exists.

Let $i\in \{1,\ldots ,k\}$. By the choice of $a_i$, we have that $a_i\notin A(P_{a_j})$ for $i<j\le k$. From the other side, $a_i\notin A(P_j)$ for $1\le j<i$, because $a_i\in R_i$ and $R_i$ does not contain the arcs of $P_a$ for $a\in R_j$ for $1\le j<i$ by the construction of the sets $R_1,\ldots ,R_k$. We obtain that the *t*-detours $P_{a_i}$ for $i\in \{1,\ldots ,k\}$ do not contain any $a_j$ for $j\in \{1,\ldots ,k\}$. By Observation 1, $H=G-\{a_1,\ldots ,a_k\}$ is a multiplicative *t*-spanner. Therefore, (*G*, *t*, *k*) is a yes-instance of Directed Multiplicative Spanner. $\square$

By Claim 1, we can apply the next rule:

#### Reduction Rule 1

If $|S|\ge \frac{1}{2}k(t+1)((k-1)t+2)$, then return a trivial yes-instance of Directed Multiplicative Spanner and stop.

From now, we assume that $|S|< \frac{1}{2}k(t+1)((k-1)t+2)$.

The analog of Reduction Rule 1 is a main step of the kernelization algorithm of Kobayashi [10] for the undirected case, because it almost immediately allows to upper bound the total number of edges of the graph. However, the directed case is more complicated, since the arcs of *t*-detours for $a\in S$ may be outside *S* contrary to the undirected case, where all the edges of *t*-detours are in cycles of length at most $t+1$ and, therefore, have *t*-detours themselves. We use the following procedure to mark the crucial arcs of potential detours.

***Marking Procedure*** Let $G'=G-S$.

(i) For every $(u,v)\in S$, find a shortest (*u*, *v*)-path *P* in $G'$ and if the length of *P* is at most *t*, then *mark* the arcs of *P*.
(ii) For every ordered pair of two distinct arcs $(u_1,v_1),(u_2,v_2)\in S$,
  - find a shortest $(u_1,u_2)$-path $P_1$ in $G'$ and if the length of $P_1$ is at most *t*, then *mark* the arcs of $P_1$,
  - find a shortest $(v_2,v_1)$-path $P_2$ in $G'$ and if the length of $P_2$ is at most *t*, then *mark* the arcs of $P_2$,
  - find a shortest $(v_1,u_2)$-path $P_3$ in $G'$ and if the length of $P_3$ is at most *t*, then *mark* the arcs of $P_3$.

Observe that marking can be done in polynomial time by Dijkstra’s algorithm. Denote by *L* the set of marked arcs. Our final rule constructs the output instance.

#### Reduction Rule 2

Consider the graph $H=(V(G),S\cup L)$. Delete the isolated vertices of *H*, and for the obtained $G^*$, output $(G^*,t,k)$.

We argue that the rule is safe.

#### Claim 2

(*G*, *t*, *k*) is a yes-instance of Directed Multiplicative Spanner if and only if $(G^*,t,k)$ is a yes-instance.

#### Proof of Claim 2

Suppose that (*G*, *t*, *k*) is a yes-instance of Directed Multiplicative Spanner. Then, by Observation 1, there are *k* distinct arcs $a_1,\ldots ,a_k\in S$ with their *t*-detours $P_1,\ldots ,P_k$, respectively, such that $a_i\notin \bigcup _{j=1}^kA(P_j)$. Notice that $a_1,\ldots ,a_k\in A(G^*)$. Consider $i\in \{1,\ldots ,k\}$ and let $a_i=(u,v)$.

Suppose that $P_i$ does not contain arcs from *S*. Then $P_i$ is a (*u*, *v*)-path in $G'=G-S$. By the first step of Marking Procedure, there is a *t*-detour $P_i'$ for $a_i$ whose arcs are in $G'$ and are marked. Then $P_i'$ is a *t*-detour for $a_i$ in $G^*$ and $a_j\notin A(P_i')$ for $j\in \{1,\ldots ,k\}$.

Assume that $P_i$ contains some arcs from *S*. Let $e_1,\ldots ,e_s$ be these arcs (in the path order with respect to $P_i$ starting from *u*). Note that $e_1,\ldots ,e_s\in A(G^*)$ and they are distinct from $a_1,\ldots ,a_k$. Let $e_j=(x_j,y_j)$ for $j\in \{1,\ldots ,s\}$. Then $P_i$ can be written as the concatenation of the paths $P_i=Q_1\circ x_1y_1\circ Q_2\circ \cdots \circ x_sy_s\circ Q_{s+1}$, where $Q_1$ is the $(u,x_1)$-subpath of $P_i$, $Q_j$ is the $(y_{j-1},x_j)$-subpath of $P_i$ for $j\in \{2,\ldots ,s\}$, and $Q_{s+1}$ is the $(y_s,v)$-subpath of $P_i$; note that some of the paths $Q_1,\ldots ,Q_{s+1}$ may be trivial, i.e., contain a single vertex. We allow trivial paths to make the notation for $P_i$ uniform. Let $j\in \{1,\ldots ,s+1\}$. If $Q_j$ is trivial, then $Q_j'=Q_j$ is a path in $G^*$, because the vertices incident to the arcs of *S* are vertices of $G^*$. Suppose that $Q_j$ is not trivial. If $j=1$, then by step (ii)(a) of Marking Procedure, there is a $(u,x_1)$-path $Q_1'$, whose arcs are in $G'$ and are marked, and the length of $Q_1'$ is at most the length of $Q_1$. For $j=s+1$, we have, by step (ii)(b), that there is a $(y_{s},v)$-path $Q_{s+1}'$, whose arcs are in $G'$ and are marked, and the length of $Q_{s+1}'$ is at most the length of $Q_{s+1}$. Suppose that $2\le j\le s$. Then by step (ii)(c), there is a $(y_{j-1},x_j)$-path $Q_j'$, whose arcs are in $G'$ and are marked, and the length of $Q_j'$ is at most the length of $Q_j$. Consider the (*u*, *v*)-walk $W_i=Q_1'\circ x_1y_1\circ Q_2'\circ \cdots \circ x_sy_s\circ Q_{s+1}'$. We have that $W_i$ is a (*u*, *v*)-walk of length at most *t* in $G^*$ such that $a_j\notin A(W_i)$ for $j\in \{1,\ldots ,k\}$. This implies that $G^*$ has a *t*-detour $P_i'$ in $G^*$ such that $a_j\notin A(P_i')$ for $j\in \{1,\ldots ,k\}$.

We obtain that for every $i\in \{1,\ldots ,k\}$, $a_i\in A(G^*)$ has a *t*-detour $P_i'$ such that $a_1,\ldots ,a_k\notin A(P_i')$. By Observation 1, we conclude that $G^*-\{a_1,\ldots ,a_k\}$ is a multiplicative spanner for $G^*$, that is, $(G^*,t,k)$ is a yes-instance of Directed Multiplicative Spanner.

For the opposite direction, assume that $(G^*,t,k)$ is a yes-instance of Directed Multiplicative Spanner. By Observation 1, there are *k* distinct arcs $a_1,\ldots ,a_k\in A(G^*)$ with their *t*-detours $P_1,\ldots ,P_k$, respectively, such that $a_i\notin \bigcup _{j=1}^kA(P_j)$. Since $G^*$ is a subgraph of *G*, $a_1,\ldots ,a_k$ have the same *t*-detours in *G*. By Observation 1, (*G*, *t*, *k*) is a yes-instance. $\square$

To upper bound the size of $G^*$, observe that Marking Procedure marks at most *t* arcs for each $a\in S$ in step (i), that is, at most |*S*|*t* arcs are marked in this step. In step (ii), we mark at most 3*t* arcs for each ordered pair of arcs of *S*. Hence, at most $3|S|(|S|-1)t$ arcs are marked in total in the second step. Since $|S|< \frac{1}{2}k(t+1)((k-1)t+2)$, we have that $G^*$ has $\mathcal {O}(k^4t^5)$ arcs. Because $G^*$ has no isolated vertices, the number of vertices is $\mathcal {O}(k^4t^5)$.

Since each of the reduction rules and Marking Procedure can be applied in polynomial time, we conclude that the total running time of our kernelization algorithm is polynomial. $\square$

### ${{\,\mathrm{\mathsf{FPT}}\,}}$ Algorithm for Directed Multiplicative Spanner

Combining Theorem 1 with the brute-force procedure that guesses *k* arcs of *G* and verifies whether the deletion of these arcs gives a multiplicative *t*-spanner, we obtain the straightforward $2^{\mathcal {O}(k\log (kt))}+n^{\mathcal {O}(1)}$ algorithm for Directed Multiplicative Spanner. If we use the intermediate steps of the kernelization algorithm, then the running time may be improved (upto some constants in the exponent) to $(kt)^{2k}\cdot n^{\mathcal {O}(1)}$. Namely, we can construct the set *S* of *t*-good arcs and execute Reduction Rule 1 of the kernelization algorithm. Then we either solve the problem or obtain an instance, where the set *S* has size at most $\frac{1}{2}k(t+1)((k-1)t+2)-1\le k^2t^2$. Then for every $R\subseteq S$ of size *k*, we check whether $G-R$ is a multiplicative *t*-spanner by computing the distances between every pair of vertices. However, we can slightly improve the parameter dependence by making use of the *random separation* technique proposed by Cai, Chan, and Chan in [3] (we refer to [5, Chapter 5] for the detailed introduction to the technique). In this subsection, we briefly sketch a Monte Carlo algorithm with false negatives for Directed Multiplicative Spanner.

#### Theorem 2

Directed Multiplicative Spanner can be solved in time $(4t)^k\cdot n^{\mathcal {O}(1)}$ by a Monte Carlo algorithm with false negatives.

#### Proof

Let (*G*, *t*, *k*) be an instance of Directed Multiplicative Spanner. If $k=0$ or $t=1$, then the problem is trivial: if $k=0$, then (*G*, *t*, *k*) is a yes-instance, and if $k>0$ and $t=1$, then (*G*, *t*, *k*) is a no-instance. From now we assume that $k\ge 1$ and $t\ge 2$.

By Observation 1, (*G*, *t*, *k*) is a yes-instance of Directed Multiplicative Spanner for (*G*, *t*, *k*) if and only if there are *k* arcs that have *t*-detours avoiding these arcs. We use random separation to distinguish the arcs that have *t*-detours and the arcs of the detours. We randomly color the arcs of *G* by two colors *red* and *blue*. An arc is colored red with probability $\frac{1}{t}$ and is colored blue with probability $\frac{t-1}{t}$. Then we try to find *k* red arcs that have *t*-detours composed by blue arcs. Let *R* be the set of arcs colored red and let *B* the set of blue arcs. For $(u,v)\in R$, it can be checked in polynomial time whether (*u*, *v*) has a *t*-detour with blue arcs by finding the distance between *u* and *v* in $G_B=(V(G),B)$. Then we greedily construct the set *S* of all red arcs with blue *t*-detours. If $|S|\ge k$, then we conclude that (*G*, *t*, *k*) is a yes-instance by Observation 1.

Suppose that (*G*, *t*, *k*) is a yes-instance of Directed Multiplicative Spanner. Then by Observation 1, there are *k* distinct arcs $a_1,\ldots ,a_k$ and their *t*-detours $P_1,\ldots ,P_k$, respectively, such that $a_1,\ldots ,a_k\notin L=\bigcup _{i=1}^kA(P_i)$. Notice that $|L|\le tk$. Then the probability that the considered random coloring colors the arcs $a_1,\ldots ,a_k$ red is $t^{-k}$ and the probability that the arcs of *L* are colored blue is at least $(\frac{t-1}{t})^{tk}$. We have that

$$\begin{aligned} \Big (\frac{t-1}{t}\Big )^t=\Big (1-\frac{1}{t}\Big )^t\ge \frac{1}{4}. \end{aligned}$$

Therefore, the probability that the arcs $a_1,\ldots ,a_k$ are red and their *t*-detours are blue is at least $(4t)^{-k}$. Respectively, the probability that the random coloring fails to color the arcs $a_1,\ldots ,a_k$ red and their *t*-detours blue is at most $1-\frac{1}{(4t)^k}$. This implies that if we iterate our algorithm for $(4t)^k$ colorings, then we either find a solution and stop or we conclude that (*G*, *t*, *k*) is a no-instance with the mistake probability at most $\Big (1-\frac{1}{ (4t)^k} \Big )^{(4t)^k}\le e^{-1}$. This gives us a Monte Carlo algorithm with running time $(4t)^k\cdot n^{\mathcal {O}(1)}$. $\square$

The same approach can be used for undirected graphs and it can be shown that Multiplicative Spanner can be solved by a Monte Carlo algorithm with false negatives in $(4t)^k\cdot n^{\mathcal {O}(1)}$ time. This improves the running time given in [10] at the cost of randomization.

The algorithm from Theorem 2 can be derandomized by using *universal sets* [15] instead of random colorings (see also  [5, Chapter 5]). However, this leads to an algorithm with worst running time that is not better than $(kt)^{2k}\cdot n^{\mathcal {O}(1)}$.

### Directed Multiplicative Spanners on Acyclic Graphs

In this section, we show that Weighted Directed Multiplicative Spanner is ${{\,\mathrm{\mathsf{FPT}}\,}}$ on DAGs when parameterized by *k* only. Formally, the problem is stated as follows: 
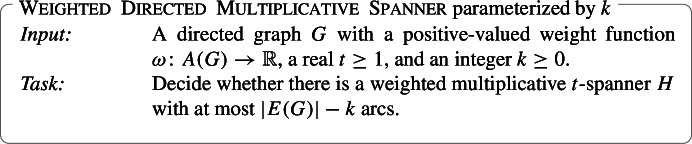


Multiplicative Spanner is know to be ${{\,\mathrm{\mathsf{NP}}\,}}$-complete for restricted graph families. However, we are not aware of hardness results for the directed variants of the problem on DAGs. Hence, we begin with showing that Directed Multiplicative Spanner and Weighted Directed Multiplicative Spanner are ${{\,\mathrm{\mathsf{NP}}\,}}$-hard on DAGs. As we are mainly interested in Parameterized Complexity, we do not try to push down the value of *t* for which Directed Multiplicative Spanner becomes ${{\,\mathrm{\mathsf{NP}}\,}}$-hard.

#### Theorem 3

Directed Multiplicative Spanner is ${{\,\mathrm{\mathsf{NP}}\,}}$-complete for every $t\ge 7$ on DAGs. Moreover, Weighted Directed Multiplicative Spanner is ${{\,\mathrm{\mathsf{NP}}\,}}$-hard for every $t>1$ when the input is restricted to DAGs.

**Fig. 1:**
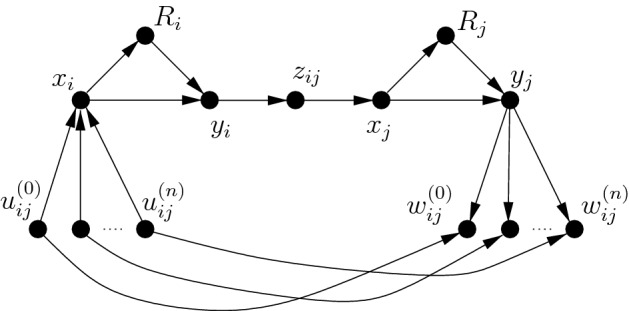
Construction of *D* for $t=7$

#### Proof

We show the theorem for Directed Multiplicative Spanner and then explain how to modify the reduction for Weighted Directed Multiplicative Spanner. We reduce from the Independent Set problem that is well-known to be ${{\,\mathrm{\mathsf{NP}}\,}}$-complete [9]. Given a graph *G* and a positive integer *k*, the problem asks whether *G* has an independent set of size at least *k*.

Let (*G*, *k*) be an instance of Independent Set and let $t\ge 7$ be an integer. Denote by $v_1,\ldots ,v_n$ the vertices of *G* and denote $m=|E(G)|$.

- For every $i\in \{1,\ldots ,n\}$, construct two vertices $x_i,y_i$, an arc $(x_i,y_i)$, and then construct a directed $(x_i,y_i)$-path $R_i$ of length $t-5$.
- For every $\{v_i,v_j\}\in E(G)$ such that $i<j$, do the following:
  - construct a vertex $z_{ij}$ and arcs $(y_i,z_{ij})$ and $(z_{ij},x_j)$,
  - construct $n+1$ vertices $u_{ij}^{(0)},\ldots ,u_{ij}^{(n)}$ and $n+1$ vertices $w_{ij}^{(0)},\ldots ,w_{ij}^{(n)}$,
  - for every $h\in \{1,\ldots ,n\}$, construct arcs $(u_{ij}^{(h)},w_{ij}^{(h)})$, $(u_{ij}^{(h)},x_{i})$, and $(y_j,w_{ij}^{(h)})$, and set $A_{ij}=\{(u_{ij}^{(0)},w_{ij}^{(0)}),\ldots ,(u_{ij}^{(n)},w_{ij}^{(n)})\}$.

Denote the obtained directed graph by *D* (see Figure 1). Clearly, *D* is a DAG. To complete the reduction, we set $k'=m(n+1)+k$. Let also $A=\bigcup _{(v_i,v_j)\in E(G),~i<j}A_{ij}$.

We claim that (*G*, *k*) is a yes-instance of Independent Set if and only if $(D,t,k')$ is a yes-instance of Directed Multiplicative Spanner.

For the forward direction, assume that $X=\{v_{i_1},\ldots ,v_{i_k}\}$ is an independent set of *G*. Consider

$$\begin{aligned} S=\{(x_{i_j},y_{i_j})\mid 1\le j\le k\}\cup A. \end{aligned}$$

Observe that $|S|=k+|A|=k+m(n+1)=k'$. We show that $H=D-S$ is a multiplicative *t*-spanner. For this, observe that every arc of *S* has a *t*-detour with its arcs in *H*. For every $j\in \{1,\ldots ,k\}$, the path $R_{i_j}$ is a *t*-detour for $(x_{i_j},y_{i_j})$. Consider an arbitrary arc $a\in A$. Then $a=(u_{ij}^{(h)},w_{ij}^{(h)})$ for some indices $i<j$ such that $\{v_i,v_j\}\in E(G)$ and some $h\in \{0,\ldots ,n\}$. Because *X* is an independent set, either $v_i\notin X$ or $v_j\notin X$. In the first case, $u_{ij}^{(h)}x_iy_iz_{ij}x_j\circ R_j\circ y_jw_{ij}^{(h)}$ has length *t* and, therefore, is a *t*-detour for *a*. Symmetrically, $u_{ij}^{(h)}x_i\circ R_i\circ y_iz_{ij} x_jy_jw_{ij}^{(h)}$ is a *t*-detour if $v_j\notin X$. We conclude that every arc of *S* has a *t*-detour in *H*. Hence, *H* is a multiplicative *t*-spanner by Observation 1.

For the opposite direction, assume that *H* is a multiplicative *t*-spanner of *D* with at most $|A(D)|-k'$ arcs. Let $S=A(D)\setminus A(H)$. Recall that every arc of *S* should have a *t*-detour in *D* by Observation 1. Then our construction implies that $S\subseteq \{(x_i,y_i)\mid 1\le i\le n\}\cup A$, because only these arcs have detours. Let $S'=S\setminus A$. Because $|S|\ge k'$, $|S'|\ge k$. Let $S'=\{(x_{i_1},y_{i_1}),\ldots ,(x_{i_s},y_{i_s})\}$ for some $s\ge k$. We show that $X=\{v_{i_1},\ldots ,v_{i_s}\}$ is an independent set of *G*. For the sake of contradiction, assume that $v_i$ and $v_j$ are adjacent in *G* for some $v_i,v_j\in X$. Consider an arc $(u_{ij}^{(h)},w_{ij}^{(h)})\in A_{ij}$ for arbitrary $h\in \{0,\ldots ,n\}$. Notice that every $(u_{ij}^{(h)},w_{ij}^{(h)})$-path *P* in *D* avoiding $(u_{ij}^{(h)},w_{ij}^{(h)})$ contains the arcs $(u_{ij}^{(h)},x_i)$ and $(y_j,w_{ij}^{(h)})$, the paths $R_i$ and $R_j$, and some $(y_i,x_j)$-path *Q*. Clearly, the length of *Q* is at least 2. Then the length of *P* is at least $2+2(t-5)+2=t+(t-6)>t$, because $t\ge 7$. This implies that $(u_{ij}^{(h)},w_{ij}^{(h)})\notin S$. Then $A_{ij}\cap S=\emptyset$ and $|A\cap S|\le (m-1)(n+1)$. Since $s\le n$, we obtain that $|S|=|S\cap A|+|S'|\le (m-1)(n+1)+n<m(n+1)\le k'$; a contradiction. This proves that *X* is an independent set of *G* and concludes the proof of the theorem for Directed Multiplicative Spanner.

For the second claim, we modify the above reduction. Let (*G*, *k*) be an instance of Independent Set and let $t'>1$. We construct the instance $(D,\omega ,t',k')$ of Weighted Directed Multiplicative Spanner as follows. First, we construct *D* for $t=7$ and define $k'$ exactly in the same way as above. Then we define the weight function $\omega (\cdot )$:

$$\begin{aligned} \omega (a)= {\left\{ \begin{array}{ll} 1+5/t',&{}\text{ if } a\in A,\\ t'/2,&{}\text{ if } a\in A(R_i)\text { for some }i\in \{1,\ldots ,n\},\\ 1,&{}\text{ otherwise }. \end{array}\right. } \end{aligned}$$

Then by the essentially the same arguments as above, one can show that (*G*, *k*) is a yes-instance of Independent Set if and only if $(D,\omega ,t',k')$ is a yes-instance of Weighted Directed Multiplicative Spanner. $\square$

Now we show the main claim of the subsection that Weighted Directed Multiplicative Spanner is ${{\,\mathrm{\mathsf{FPT}}\,}}$ on DAGs when parameterized by *k* only.

#### Theorem 4

Weighted Directed Multiplicative Spanner can be solved in $k^{2k}\cdot n^{\mathcal {O}(1)}$ time on DAGs.

#### Proof

Let $(G,\omega ,t,k)$ be an instance of Weighted Directed Multiplicative Spanner. Consider the set *S* of arcs of *G* having *t*-detours. For every $a\in S$, denote by $P_a$ an arbitrary *t*-detour for *a*.

Let $a_1,a_2\in S$ be distinct arcs, and let $a_1=(u_1,v_1)$ and $a_2=(u_2,v_2)$. Assume that *G* has a path *P* such that $a_1,a_2\in A(P)$. We claim that $a_2\notin A(P_{a_1})$ and $a_1\notin A(P_{a_2})$. To show this, assume that $a_1$ occurs in *P* before $a_2$. Then $u_1\prec v_1\preceq u_2\prec v_2$ with respect to an arbitrary topological ordering of the vertices of *D*. Suppose that $a_2\in A(P_{a_1})$. Then $P_{a_1}$ has the $(v_2,v_1)$-subpath *Q*. However, this contradict that $v_1\prec v_2$. Symmetrically, if $a_1\in A(P_{a_2})$, then $P_{a_2}$ has the $(u_2,u_1)$-subpath contradicting that $u_1\prec u_2$. This proves the claim.

Using the above claim, we now show that if $|S|> k(k-1)$, then $(G,\omega ,t,k)$ is a yes-instance. If there exists an arc $a\in S$ such that the set $S'=A(P_a)\cap S$ contains at least *k* arcs, then $G-S'$ is a multiplicative *t*-spanner by Observation 1. Indeed, because the arcs of $S'$ are on the same path $P_a$, the detours $P_e$ for $e\in S'$ do not contain any arc of $S'$ from the previous claim. In the other case, $|A(P_a)\cap S|\le k-1$ for every $a\in S$. Clearly, $|(A(P_a)\cup \{a\})\cap S|\le k$ for every $a\in S$. Then because $|S|> k(k-1)$, we can greedily select *k* distinct arcs $a_1,\ldots ,a_k\in S$ such that the sets $(A(P_{a_i})\cup \{a_i\}) \cap S$ for $i\in \{1,\ldots ,k\}$ are disjoint. We choose $a_1\in S$ arbitrarily. Let $2\le i\le k$ and assume that $a_1,\ldots ,a_{i-1}$ are selected. Because $|S|> k(k-1)$ and $|(A(P_{a_j})\cup \{a_j\})\cap S|\le k$ for every $j\in \{1,\ldots ,i-1\}$, we have that $|S\setminus \bigcup _{j=1}^{i-1}(A(P_{a_j})\cup \{a_j\})|>k(k-1)-k(i-1)\ge 0$. Then we arbitrary choose $a_i\in S\setminus \bigcup _{j=1}^{i-1}(A(P_{a_j})\cup \{a_j\})$. This way we construct $a_1,\ldots ,a_k$. Then we conclude that $G-\{a_1,\ldots ,a_k\}$ is a multiplicative *t*-spanner from Observation 1.

This leads to the following algorithm for Weighted Directed Multiplicative Spanner. First, we construct *S*. Clearly, it can be done in polynomial time by Dijkstra’s algorithm. If $|S|> k(k-1)$, then we return the answer yes. Otherwise, we consider all subsets $R\subseteq S$ of size *k*, and for each *R*, we check whether $G-R$ is a multiplicative *t*-spanner. This can be done by making use of Observation 1 and the Dijkstra’s algorithm. The algorithm returns yes if $G-R$ is a multiplicative *t*-spanner. We return no if we fail to find a spanner this way. Since there are at most $$\left( {\begin{array}{c}k(k-1)\\ k\end{array}}\right) $$ sets *R*, the algorithm runs in $k^{2k}\cdot n^{\mathcal {O}(1)}$ time. This concludes the proof. $\square$

Note that unlike Directed Multiplicative Spanner, the arguments of Theorem 4 do not yield a kernel because even though the weight of *t*-detours of the arcs of *S* is bounded, their lengths could be very long because the weights are real numbers.

## Directed Additive *t*-spanners

In this section, we consider Directed Additive Spanner and show that the problem is hard on DAGs for every $t\ge 1$.

### Theorem 5

Directed Additive Spanner is ${{\,\mathrm{\mathsf{W}}\,}}[1]$-hard on DAGs when parameterized by *k* for every $t\ge 1$.

**Fig. 2:**
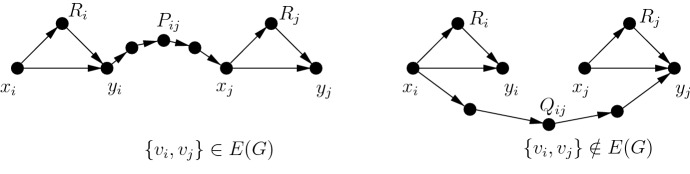
Construction of *D* for $t=1$

### Proof

We reduce from the Independent Set problem. Recall that, given a graph *G* and a positive integer *k*, the problem asks whether *G* has an independent set of size at least *k*. Independent Set parameterized *k* is well-known to be one of the basic ${{\,\mathrm{\mathsf{W}}\,}}[1]$-complete problems (see [5, 6]).

Let (*G*, *k*) be an instance of Independent Set and let *t* be a positive integer. Denote by $v_1,\ldots ,v_n$ the vertices of *G*.

- For every $i\in \{1,\ldots ,n\}$, construct two vertices $x_i,y_i$, an arc $(x_i,y_i)$, and then construct a directed $(x_i,y_i)$-path $R_i$ of length $t+1$.
- For every $i,j\in \{1,\ldots ,n\}$ such that $i<j$, do the following:
  - if $\{v_i,v_j\}\in E(G)$, then construct a directed $(y_i,x_j)$-path $P_{ij}$ of length $t+3$,
  - if $\{v_i,v_j\}\notin E(G)$, then construct a directed $(x_i,y_j)$-path $Q_{ij}$ of length $t+3$.

Denote the obtained directed graph by *D* (see Figure 2). It is straightforward to verify that *D* is a DAG. We show that (*G*, *k*) is a yes-instance of Independent Set if and only if (*D*, *t*, *k*) is a yes-instance of Directed Additive Spanner.

Suppose that $I=\{v_{i_1},\ldots ,v_{i_k}\}$ is an independent set of size *k* in *G*. Let $S=\{(x_{i_1},y_{i_1}),\ldots ,(x_{i_k},y_{i_k})\}$. We show that $D'=D-S$ is an additive *t*-spanner for *D*.

We first claim that for every two vertices *u* and *w* of *D*, each shortest (*u*, *w*)-path in *D* contains at most one arc of *S*. The proof is by contradiction. Assume that there are $u,w\in V(D)$ and a shortest (*u*, *w*)-path *P* such that *P* contains at least two arcs of *S*. Let $(x_i,y_i)$ and $(x_j,y_j)$ be such arcs and let $i<j$. By the construction, $(x_i,y_i)$ occurs before $(x_j,y_j)$ in *P*. Since the arcs of *S* correspond to vertices of the independent set *I*, $v_i$ and $v_j$ are not adjacent in *G*. Therefore, *D* contains the $(x_i,y_j)$-path $Q_{ij}$ of length $t+3$. Since *P* is a shortest path containing $(x_i,y_i)$ and $(x_j,y_j)$, the $(y_i,x_j)$-subpath of *P* should have length at most $t+1$. However, by the construction, the distance between $y_i$ and $x_j$ is at least $t+3$; a contradiction proving the claim.

Now let *u* and *w* be two vertices of *D*. Let *P* be a shortest (*u*, *w*)-path in *D*. If *P* is a path in $D'$, then ${{\text {dist}}}_{D'}(u,w)={{\text {dist}}}_D(u,w)$. Suppose that *P* is not a path in $D'$. Then *P* contains a unique arc $(x_i,y_i)\in S$ by the proved claim. Let $P_1$ be the $(u,x_i)$-subpath of *P* and let $P_2$ be the $(y_i,w)$-subpath. Let $P'=P_1\circ R_i \circ P_2$. Observe that $P'$ is a path in $D'$. Since the length of $P'$ is the length of *P* plus the length of $R_i$, that is, $t+1$, ${{\text {dist}}}_{D'}(u,w)\le {{\text {dist}}}_D(u,w)+t$. This implies that $D'$ is an additive *t*-spanner of *D*.

Now we assume that (*D*, *t*, *k*) is a yes-instance of Directed Additive Spanner. Then there is a set of *k* arcs $S\subseteq A(D)$ such that $D'=D-S$ is an additive *t*-spanner. Observe that if $(u,v)\in S$, then *D* has an (*u*, *v*)-path *P* that does not use the arc (*u*, *v*). Otherwise, ${{\text {dist}}}_{D'}(u,v)=+\infty$ and ${{\text {dist}}}_{D'}(u,v)>{{\text {dist}}}_D(u,v)+t$. Therefore, $S\subseteq \{(x_1,y_1),\ldots ,(x_n,y_n)\}$. Let $S=\{(x_{i_1},y_{i_1}),\ldots ,(x_{i_k},y_{i_k})\}$. We claim that $I=\{v_{i_1},\ldots ,v_{i_k}\}$ is an independent set of *G*. Assume, for the sake of contradiction, that this is not the case and there are $v_i,v_j\in I$ such that $v_i$ and $v_j$ are adjacent in *G*. Let $i<j$. Consider the vertices $x_i$ and $y_j$ of *D*. Since $\{v_i,v_j\}\in E(G)$, $P=x_iy_i\circ P_{ij}\circ x_jy_j$ is an $(x_i,y_j)$-path of length $t+5$, that is, ${{\text {dist}}}_D(x_i,y_j)\le t+5$. The path $P'=R_i\circ P_{ij}\circ R_j$ has length $3t+5$ and is a path in $D'$. Any other $(x_i,y_j)$-path in $D'$ uses at least two paths of length $t+3$: one of the paths $P_{ii'}$ and $Q_{ii'}$ for some $i'\in \{1,\ldots ,n\}$ such that $i'\ne j$, and one of the paths $P_{j'j}$ and $Q_{j'j}$ for some $j'\in \{1,\ldots ,n\}$ such that $j'\ne i$. This means that ${{\text {dist}}}_{D'}(x_i,y_j)\ge 2(t+3)>(t+5)+t\ge {{\text {dist}}}_{D}(x_i,y_j)+t$ contradicting that $D'$ is an additive *t*-spanner. We conclude that *I* is an independent set of *G* and, therefore, (*G*, *k*) is a yes-instance of Independent Set. $\square$

## Conclusion

We proved that Directed Multiplicative Spanner admits a kernel of size $\mathcal {O}(k^4t^5)$ and can be solved in $(4t)^k\cdot n^{\mathcal {O}(1)}$ randomized time. We also demonstrated that (Weighted) Directed Multiplicative Spanner is ${{\,\mathrm{\mathsf{NP}}\,}}$-complete on DAGs and can be solved in $k^{2k}\cdot n^{\mathcal {O}(1)}$ on this class of directed graphs. This leads to the question whether Multiplicative Spanner is FPT on general graphs when parameterized by *k* only for both undirected and directed cases. Also, is the weighted version of Multiplicative Spanner ${{\,\mathrm{\mathsf{FPT}}\,}}$ when parameterized by *k* and *t* on general graphs? Again, this question is open for both undirected and directed graphs.

Further we proved that Directed Additive Spanner is ${{\,\mathrm{\mathsf{W}}\,}}[1]$-hard for every fixed $t\ge 1$ even if the input graphs are restricted to DAGs. This result leads to the question whether Directed Additive Spanner is tractable on some special classes of directed graphs, like planar directed graphs. We believe that this problem may be interesting even if the distortion parameter *t* is assumed to be a constant.

Another possible direction of research is considering different types of directed graph spanners. For example, what can be said about the roundtrips spanners introduced by Roditty, Thorup, and Zwick [18]? A spanning subgraph *H* of a directed graph *G* is a multiplicative *t-roundtrip-spanner* if for every two vertices *u* and *v*, ${{\text {dist}}}_{H}(u,v)+{{\text {dist}}}_H(v,u)\le t({{\text {dist}}}_G(u,v)+{{\text {dist}}}_G(v,u))$, that is, *H* approximates the sum of the distances between any two vertices in both directions. Is the analog of Directed Multiplicative Spanner for roundtrip spanners ${{\,\mathrm{\mathsf{FPT}}\,}}$? Notice that we cannot use Observation 1 that is crucial for our results for the new problem. Consider, for example, the directed graph *G* constructed as follows: construct two vertices *u* and *v* and an arc (*u*, *v*), and then add a (*u*, *v*)-path $P_1$ and a (*v*, *u*)-path $P_2$ of arbitrary length $\ell \ge 2$ that are internally vertex disjoint. Then it is easy to see that $H=G-(u,v)$ is a 2-roundtrip spanner for *G*. However, *H* has no short detour for (*u*, *v*). It is also possible to define additive *t*-roundtrip-spanners and consider the analog of Directed Additive Spanner. We conjecture that this problem is at least as hard as Directed Additive Spanner.
